# Photobiomodulation drives pericyte mobilization towards skin regeneration

**DOI:** 10.1038/s41598-020-76243-7

**Published:** 2020-11-06

**Authors:** Isabella Bittencourt do Valle, Pedro Henrique Dias Moura Prazeres, Ricardo Alves Mesquita, Tarcília Aparecida Silva, Hortência Maciel de Castro Oliveira, Pollyana Ribeiro Castro, Iuri Dornelas Prates Freitas, Sicília Rezende Oliveira, Natália Aparecida Gomes, Rafaela Férrer de Oliveira, Larissa Fassarela Marquiore, Soraia Macari, Flávio Almeida do Amaral, Humberto Jácome-Santos, Lucíola Silva Barcelos, Gustavo Batista Menezes, Márcia Martins Marques, Alexander Birbrair, Ivana Márcia Alves Diniz

**Affiliations:** 1 Department of Restorative Dentistry, School of Dentistry, Universidade Federal de Minas Gerais, Av. Antônio Carlos, 6627, Pampulha, Belo Horizonte, MG 31.270-901 Brazil; 2 Department of Oral Pathology and Surgery, School of Dentistry, Universidade Federal de Minas Gerais, Belo Horizonte, Minas Gerais Brazil; 3 Departament of Pathology, Biological Sciences Institute, Universidade Federal de Minas Gerais, Belo Horizonte, Minas Gerais Brazil; 4 Department of Morphology, Biological Sciences Institute, Universidade Federal de Minas Gerais, Belo Horizonte, Minas Gerais Brazil; 5 Department of Physiology and Biophysics, Biological Sciences Institute, Universidade Federal de Minas Gerais, Belo Horizonte, Minas Gerais Brazil; 6 School of Dentistry, Faculdade Sete Lagoas, Sete Lagoas, Minas Gerais Brazil; 7 Department of Biochemistry and Immunology, Biological Sciences Institute, Universidade Federal de Minas Gerais, Belo Horizonte, Minas Gerais Brazil; 8 Ibirapuera University, São Paulo, São Paulo Brazil

**Keywords:** Regeneration, Optics and photonics

## Abstract

Photobiomodulation is being widely applied for improving dermal or mucosal wound healing. However, the underlying cellular and molecular processes that directly contribute to its effects remain poorly understood. Pericytes are relevant cells involved in the wound microenvironment and could be one of the main targets of photobiomodulation due to their plasticity and perivascular localization. Herein, we investigate tissue repair under the photobiomodulation stimulus using a pericyte labeled (or reporter) transgenic mice. Using a model of two contralateral back wounds, one the control and the other photoactivated daily (660 nm, 20 mW, 0.71 W/cm^2^, 5 J/cm^2^, 7 s, 0.14 J), we showed an overall influx of immune and undifferentiated cells and higher mobilization of a potent pericyte subpopulation (Type-2 pericytes) in the photoactivated wounds in comparison to the controls. Doppler analysis showed a significant increase in the blood flow in the photoactivated wounds, while marked vascular supply was observed histologically. Histochemical analysis has indicated more advanced stages of tissue repair after photoactivation. These data suggest that photobiomodulation significantly accelerates tissue repair through its vascular effects with direct recruitment of pericytes to the injury site.

## Introduction

Wounds induced by burns, chronic disorders, and in consequence of radiotherapy/chemotherapy treatments affect millions of people. Delayed or aberrant wounds result in quality of life impairment and long hospitalization periods, leading to high treatment costs and overloaded health systems^1^. Accumulating scientific evidence suggests that photobiomodulation (PBM) therapy is potentially useful in medical applications. PBM is particularly attractive in superficial injuries—where it can be readily accessible to a light source—and modulate pain, inflammatory and immune processes, being recognized as a powerful tool in wound healing and tissue repair^2–7^.

The mechanisms underlying PBM therapy are mostly related to the generation of energy and reactive oxygen species in cells and tissues, thus modulating cell signaling and metabolism in an injured milieu. PBM is also known to activate critical endogenous molecules such as Fibroblast Growth Factor, Vascular Endothelial Growth Factor, Bone Morphogenetic Protein 4, Transforming Growth Factor β, and NF-κB^3,5,8–10^ and is recognized to stimulate neovascularization^11–13^. However, the lack of understanding of the primary cellular and molecular components involved in the photoactivation process hinders adequate evidence of the therapy and its broader application.

Among the cells with relevant participation in the regeneration of cutaneous wounds are the pericytes. These cells are recognized as elemental constituents of the crosstalk among cells in the perivascular space^14^. Pericytes communication network system especially involves endothelial cells, in which they interplay with by physical contact and by paracrine signaling^15^. For instance, these cells are responsible for vessel stabilization^16^, blood flow regulation^17^ and may present functions similar as mesenchymal stem cells^18–20^ generating other cell populations, such as myogenic, adipogenic, angiogenic, and neurogenic progenitors^21,22^. Two pericytes subpopulations were described in the skeletal muscle interstice and other organs, the Type-1 (Nestin GFP^-^/NG2 DsRed^+^) and the Type-2 (Nestin GFP^+^/NG2 DsRed^+^)^23,24^. Type-2 pericytes can differentiate into the neural lineage and skeletal muscle and blood vessel tissues, while Type-1 pericytes mostly contribute to adipose tissue and fibrosis formation^21,23^.

The relationship between pericytes recruitment and PBM is poorly known. Pericytes could be one of the PBM's main targets due to their incredible versatility and close connection to the tissue microvasculature. Using pericyte labeled (or reporter) transgenic mice (Nestin-GFP^+^/NG2-DsRed^+^) model, we investigate PBM's effect during tissue repair. In parallel, this model also allowed the observation of Nestin-expressing progenitors or undifferentiated cells. Herein, we demonstrate that PBM induced significant vascular changes and directed the mobilization of undifferentiated and immune cells, but particularly pericytes, into the injury site, thus modulating their responses in favor of tissue repair.

## Results

### Increased number of Type-2 pericytes and undifferentiated cells were mobilized in PBM-treated wound edges in the initial experimental times

**Figure 1:**
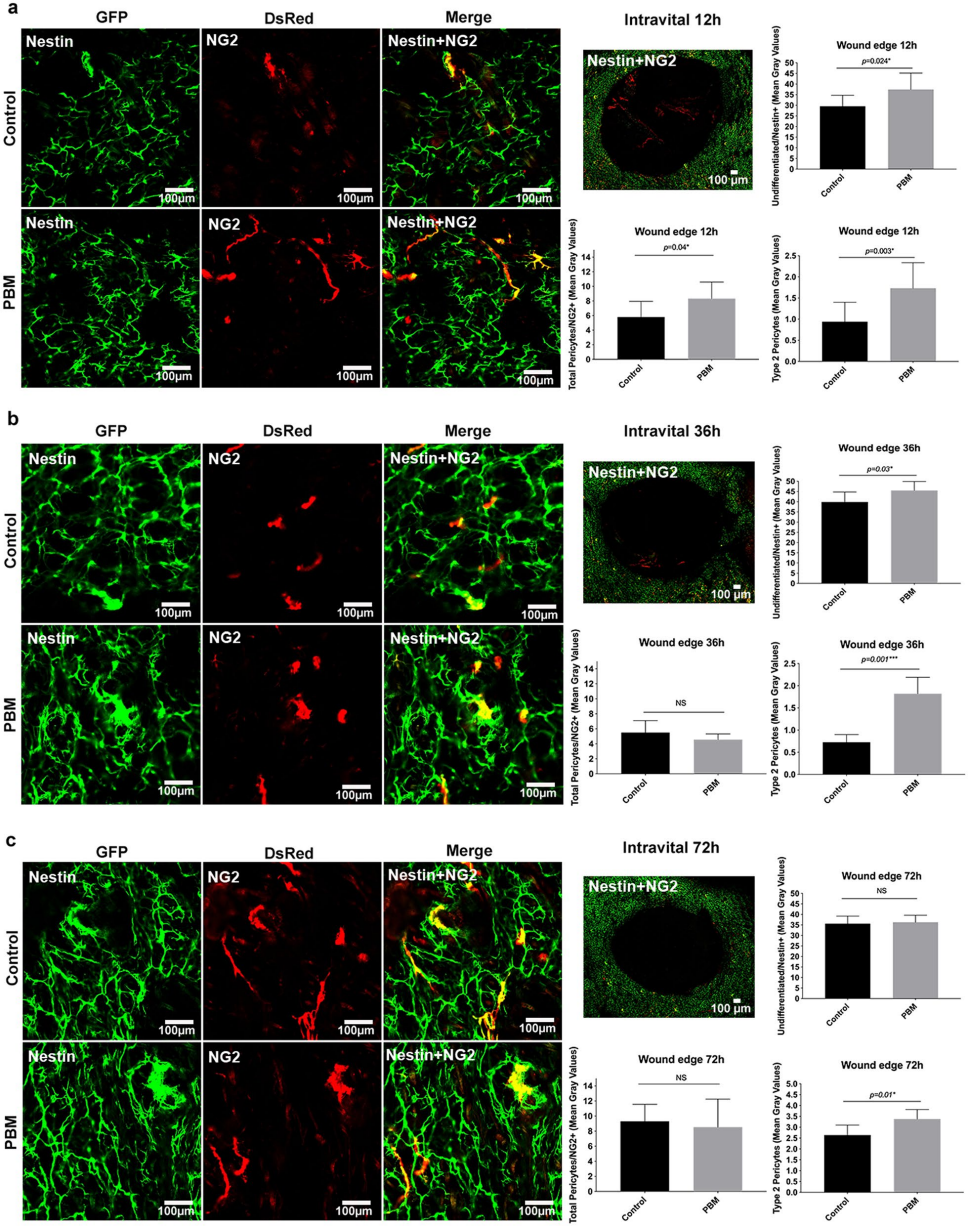
Intravital evaluation of pericytes recruitment after photobiomodulation therapy. Intravital confocal microscopy analysis after 12 h (**a**), 36 h (**b**), and 72 h (**c**) in the edges of the PBM-treated and control wounds. A representative epifluorescence scan large image (10 ×) of the total wound area of PBM-treated wounds is depicted at each experimental time. Bar graphs show undifferentiated/Nestin^+^ cells, total pericytes/NG2^+^, and Type-2 pericytes in yellow/NG2^+^Nestin^+^ (mean fluorescence) quantification. Data are shown as the mean ± SD, n = 5 for each group. *N.S.* non-significant. Scale bar = 100 µm.

To observe undifferentiated (Nestin^+^), pericytes total population (NG2^+^) and Type-2 pericytes subset (Nestin^+^NG2^+^) at the edge of the wounds artificially produced by biopsy punches (Sup. Figs. S1, S2), we performed an intravital analysis in the Nestin^+^/NG2^+^ transgenic mice 12 h, 36 h and 72 h after PBM (Fig. 1). At the 12 h time interval after the first photoactivation, the edges of PBM-treated wounds showed an increased amount of undifferentiated cells (Nestin^+^; *p* = 0.024), total pericytes population (NG2^+^; *p* = 0.040) and Type-2 pericytes subset (Nestin^+^, NG2; *p* = 0.003) when compared to the edges of the control wounds (Fig. 1a). Twelve hours after the second irradiation (i.e., at the 36 h time interval), the counts of undifferentiated cells and Type-2 pericytes were still higher in the PBM-treated wounds concerning the control groups (*p* = 0.030 and *p* = 0.001*,* respectively), despite no difference was seen in total pericytes population (*p* = 0.212) (Fig. 1b). At the 72 h time interval, the intravital analysis of the edges of the wounds showed that the Type-2 pericytes subset were still higher in the PBM-treated wounds than in control (*p* = 0.010); however, there were no differences in the number of undifferentiated cells and total pericytes population between PBM-treated and control wounds (*p* = 0.714 and *p* = 0.629, respectively) (Fig. 1c).

### PBM promoted augmented blood flow and more uniform closure of the wounds

**Figure 2:**
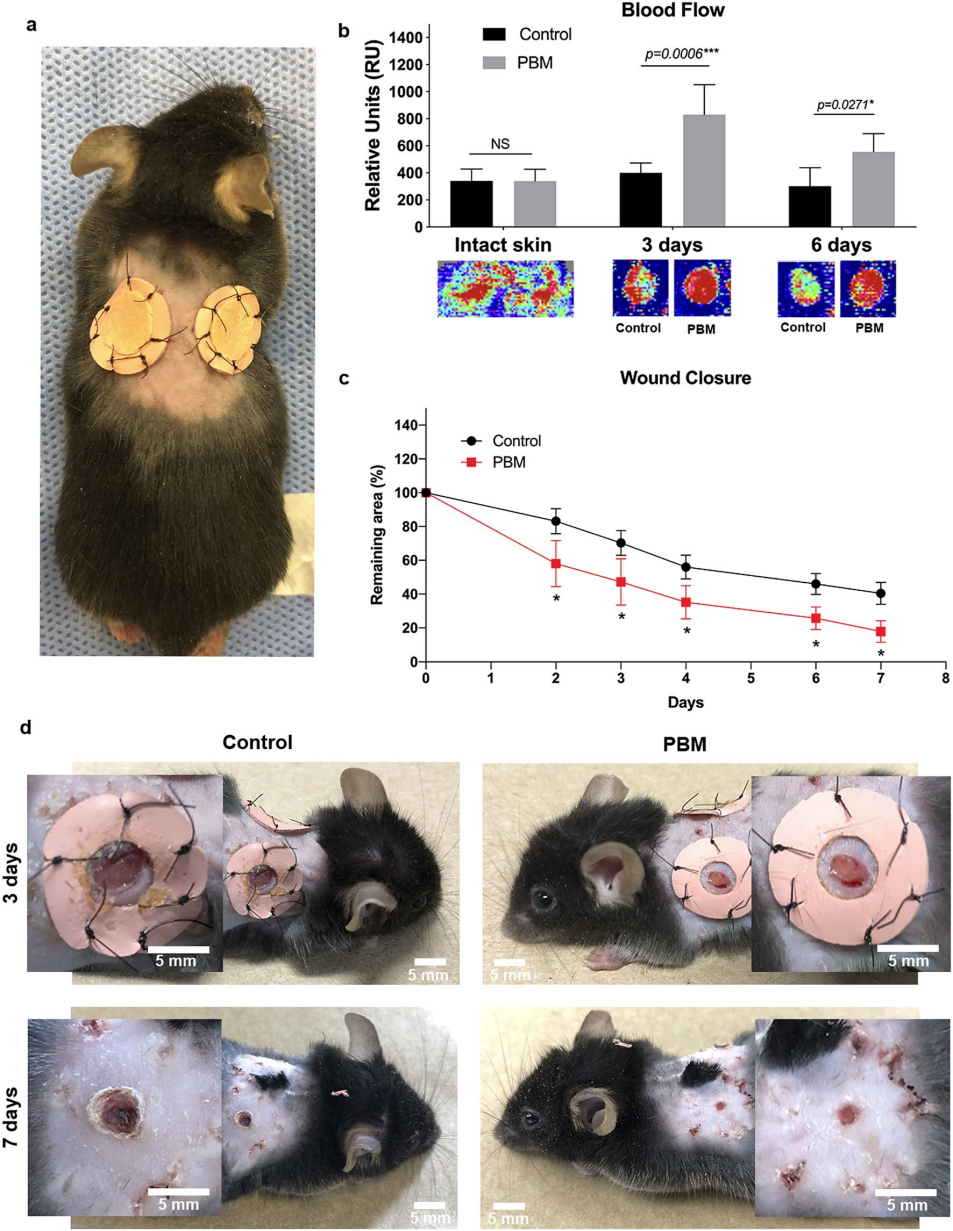
Healing pattern of the wounded skin over time. Skin wound healing model (**a**). Blood flow evaluation in control and PBM-treated wounds at baseline, day 3, and day 6 post-surgery. Red colors below the bars represent greater blood flow, while turbulent flow is represented in yellow or green (**b**). Remaining wound area (%) for total closure from day 2 until day 7 (**c**). Macroscopic appearance of control and PBM-treated wounds at days 3 and 7. Note that PBM-treated wounds are humid and with less rolled edges—3rd day; smaller, more uniformly healed and thinner eschar than the control at day 7 (**d**). Data are shown as the mean ± SD, n = 5 for each group. N.S. = non-significant. Scale bar = 5 mm.

Figure 2 depicts the blood flow and macroscopic analysis of the two bilateral wounds produced in the mice (Fig. 2a). Accordingly, the same mouse served as its control. Doppler blood flow analysis demonstrated a significant increase in the blood flow toward the wound in the PBM-treated groups (*p* < 0.001 and *p* = 0.0271), regardless of the experimental time (3 or 6 days after the first photoactivation) compared to the control groups (Fig. 2b). Moreover, PBM-treated wounds have shown a progressive and significant reduction of the wound size at all experimental times concerning the respective controls (*p* = 0.015) (Fig. 2c). After 3 days, PBM-treated wounds were qualitatively more humid and less rolled than the controls (Fig. 2d). At the last experimental time (7 days), PBM-treated groups presented qualitatively more uniform and concentric closure of the wounds (Fig. 2d). Also, the photoactivated wounds showed thinner eschars concerning the control groups (Fig. 2d).

### Type-2 pericytes and undifferentiated cells accumulated in the dermal region of the wounds upon PBM

**Figure 3:**
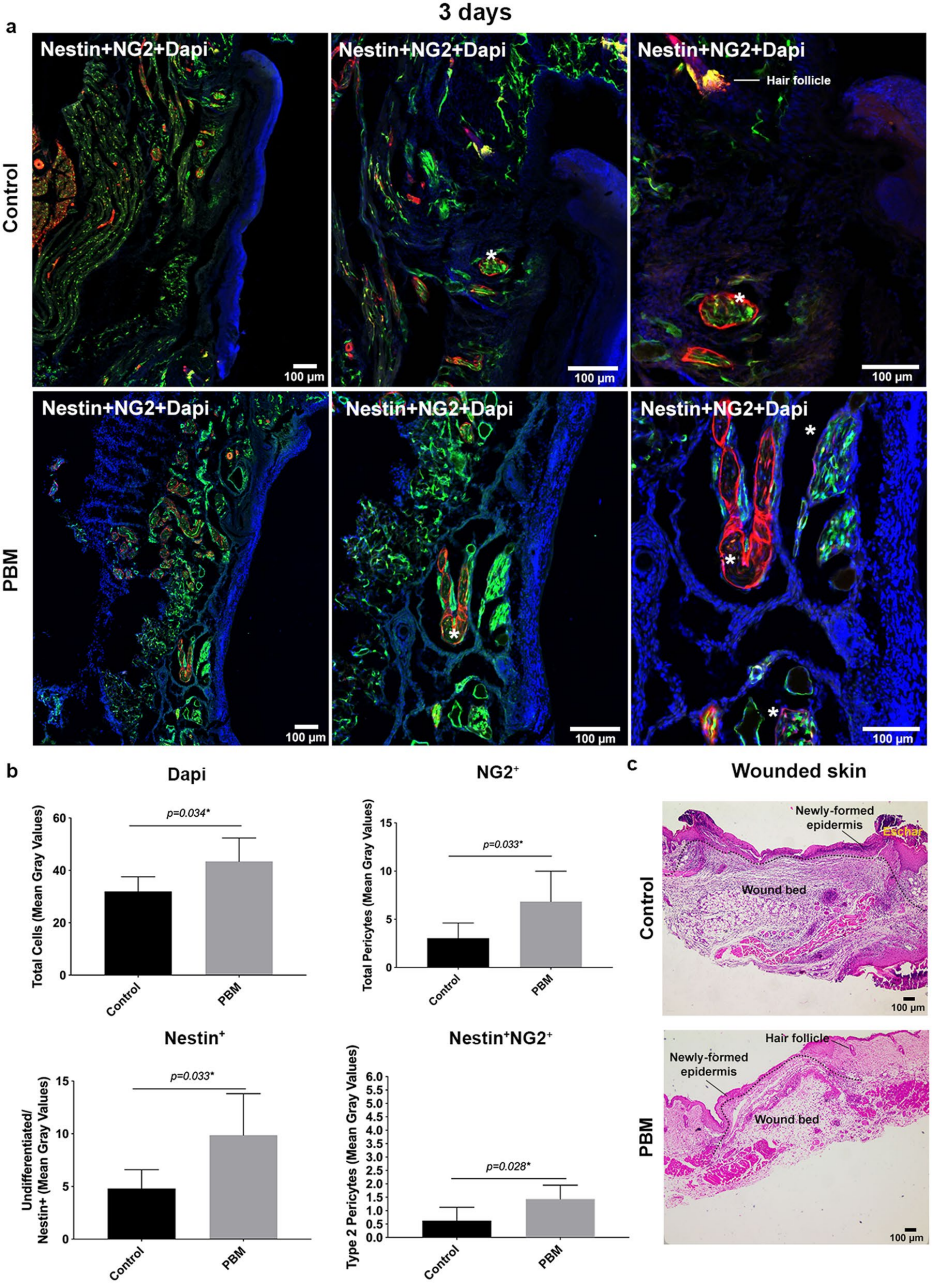
Labeled cell tracking and characterization in transversal sections at day 3 post-surgery. Representative epifluorescence merged images of control and PBM-treated wounds showing tissue morphology and labeled-cells distribution in both groups. White asterisks are showing structures suggestive of blood vessels with cells inside (**a**). Bar graphs showing the total cells (DAPI—blue), total pericytes (NG2^+^), undifferentiated cells (Nestin^+^), and Type-2 pericytes (Nestin^+^NG2^+^—yellow) (mean fluorescence) quantification (**b**). Hematoxylin and eosin-stained (H&E) sections of the full-thickness excisional wound of both groups at day 3 post-surgery (**c**). Data are shown as the mean ± SD, n = 5 for each group. Scale bar = 100 µm.

**Figure 4:**
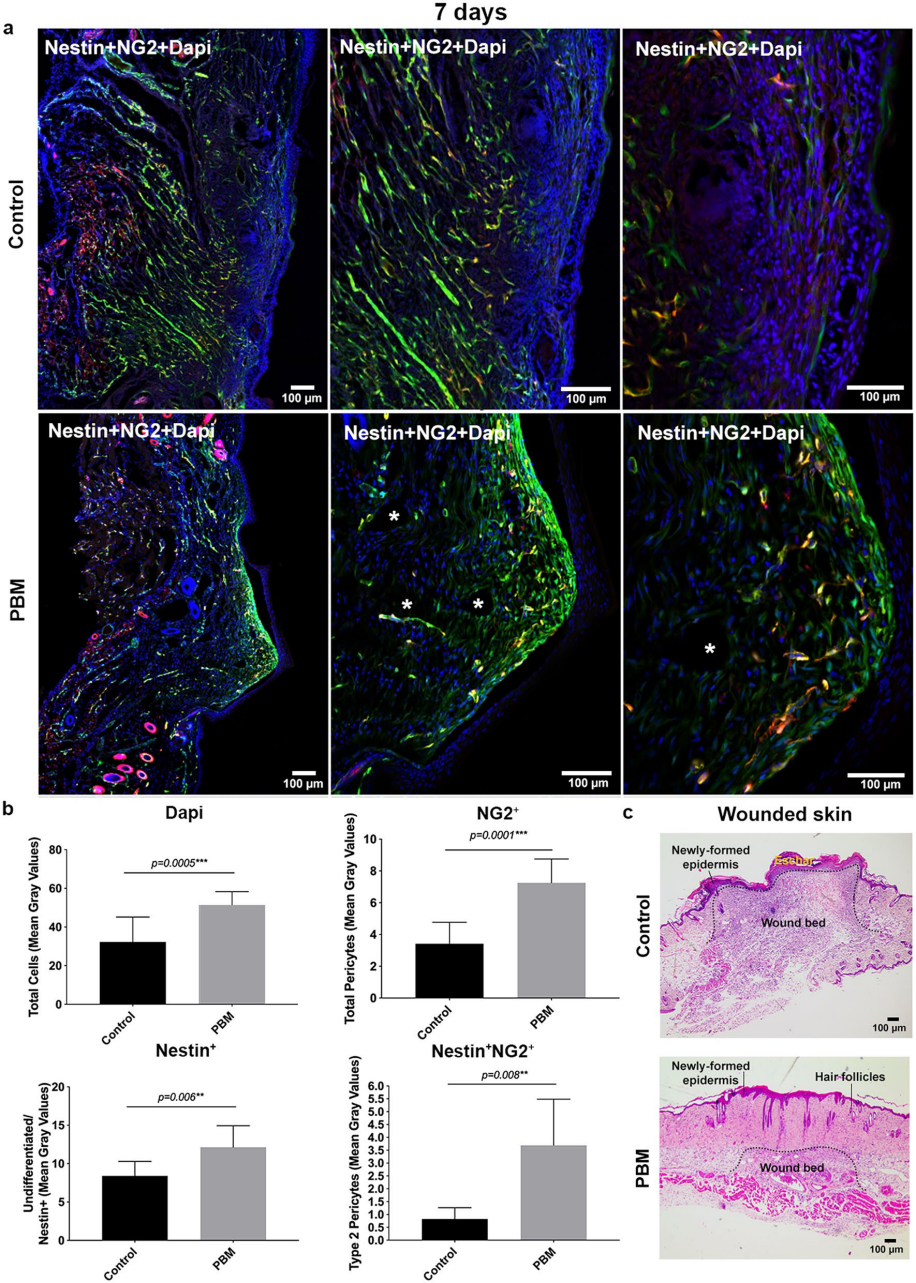
Labeled cell tracking and characterization in transversal sections at day 7 post-surgery. Representative epifluorescence merged images of control and PBM-treated wounds showing tissue morphology and labeled-cells distribution in both groups. White asterisks are showing empty structures suggestive of blood vessels (**a**). Bar graphs showing the total cells (DAPI—blue), total pericytes (NG2^+^), undifferentiated cells (Nestin^+^), and Type-2 pericytes (Nestin^+^NG2^+^—yellow) (mean fluorescence) quantification (**b**). H&E-stained sections of the full-thickness excisional wound of both groups at day 7 post-surgery (**c**). Data are shown as the mean ± SD, n = 5 for each group. Scale bar = 100 µm.

Samples at 12 h (Sup. Fig. S3), day 3 (Fig. 3) and 7 (Fig. 4) post-surgery were transversally sectioned and observed by confocal microscopy in order to verify the colocalization of labeled cells with Dapi during the wound healing process. Undifferentiated cells (Nestin^+^), total pericytes population (NG2^+^) and Type-2 pericytes subpopulation (Nestin^+^, NG2^+^, and Dapi colocalization) have accumulated more in the photoactivated wounds than in the controls on the 3rd day of treatment (*p* = 0.033; *p* = 0.033 and *p* = 0.028, respectively) (Fig. 3a,b). These cells were observed inside and around vessels and frequently seemed to be detaching from them (Fig. 3a). The NG2^+^ cells were mainly found in delimiting these vessels. Total cell quantification (Dapi fluorescence) was significantly higher in the PBM-treated wounds, meaning more significant input of cells in the wounds under the PBM stimulus than in the controls (*p* = 0.034) (Fig. 3b). The same features were observed in the samples analyzed at the 7 day-time interval (Fig. 4a,b). Otherwise, the NG2^+^ cells were no longer seen around the vessels but were dispersed in the wound bed in the dermis region in both groups (Fig. 4a). In the photoactivated wounds, the cells were clearly attracted to the wound bed’s upper dermal area, immediately underneath the epithelium (Fig. 4a). Additionally, at the 3rd and 7th-day post-surgery, the NG2^+^ and Nestin^+^/NG2^+^ cells appeared colocalized with Dapi (pinkish and yellowish components, respectively) in areas correspondent to the bulge region of the hair bulb (Sup. Fig. S4). Sometimes, these cells looked to be detaching from those skin appends (Sup. Fig. S4).

### PBM has stimulated angiogenesis and promoted advanced tissue repair

**Figure 5:**
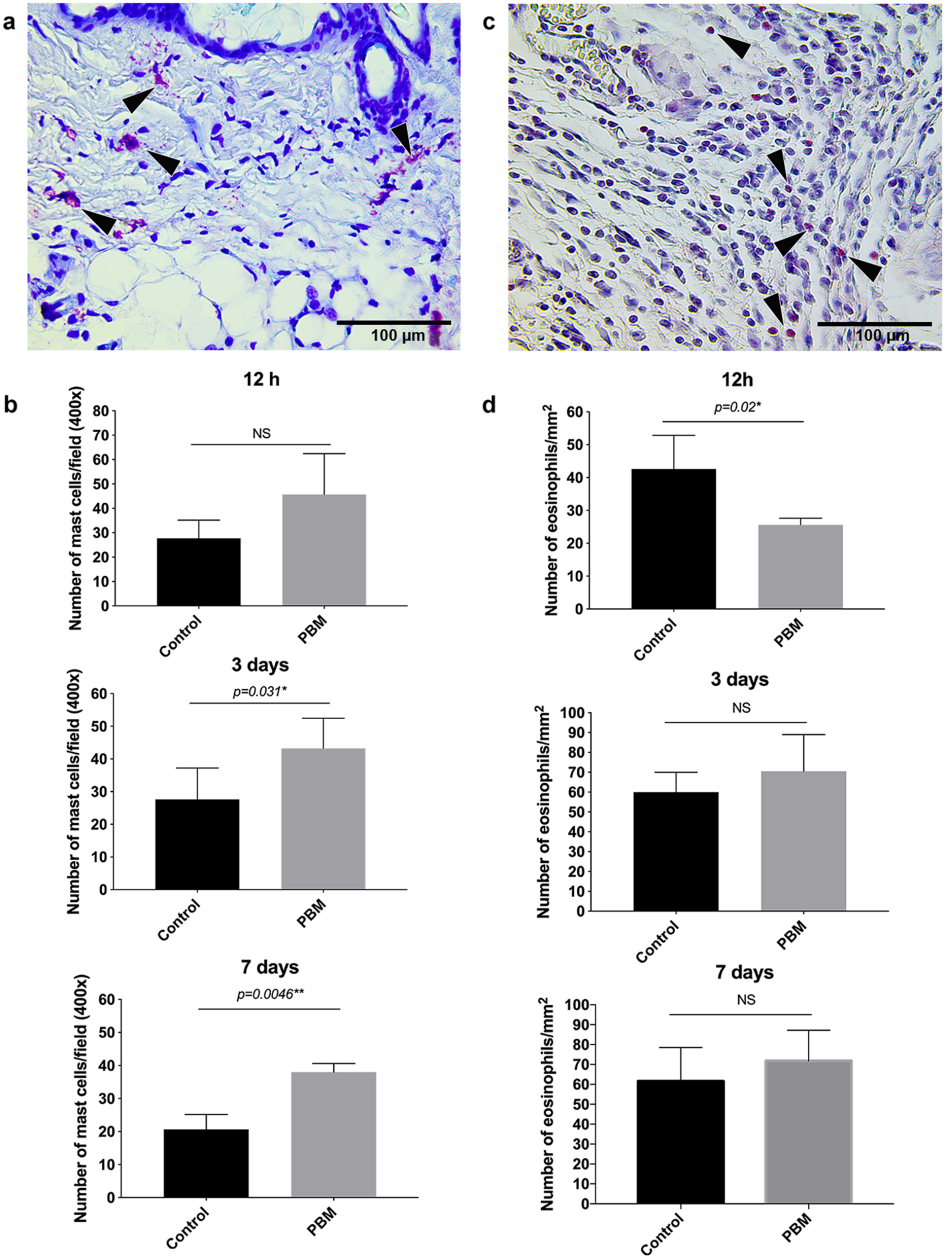
Number of mast cells and eosinophils in response to PBM therapy. Representative Toluidine Blue O (**a**) or Sirius Red (**c**) stained sections of the full-thickness excisional wound showing mast cells and eosinophils, respectively (both from PBM-treated samples at day 3 post-surgery). Bar graphs showing cellular quantification of mast cells (**b**) and eosinophils (**d**) in control and PBM-treated groups at 12 h, day 3 , and 7 post-surgery. Data are shown as the mean ± SD, n = 5 for each group. *N.S.* non-significant. Scale bar = 100 µm.

**Figure 6:**
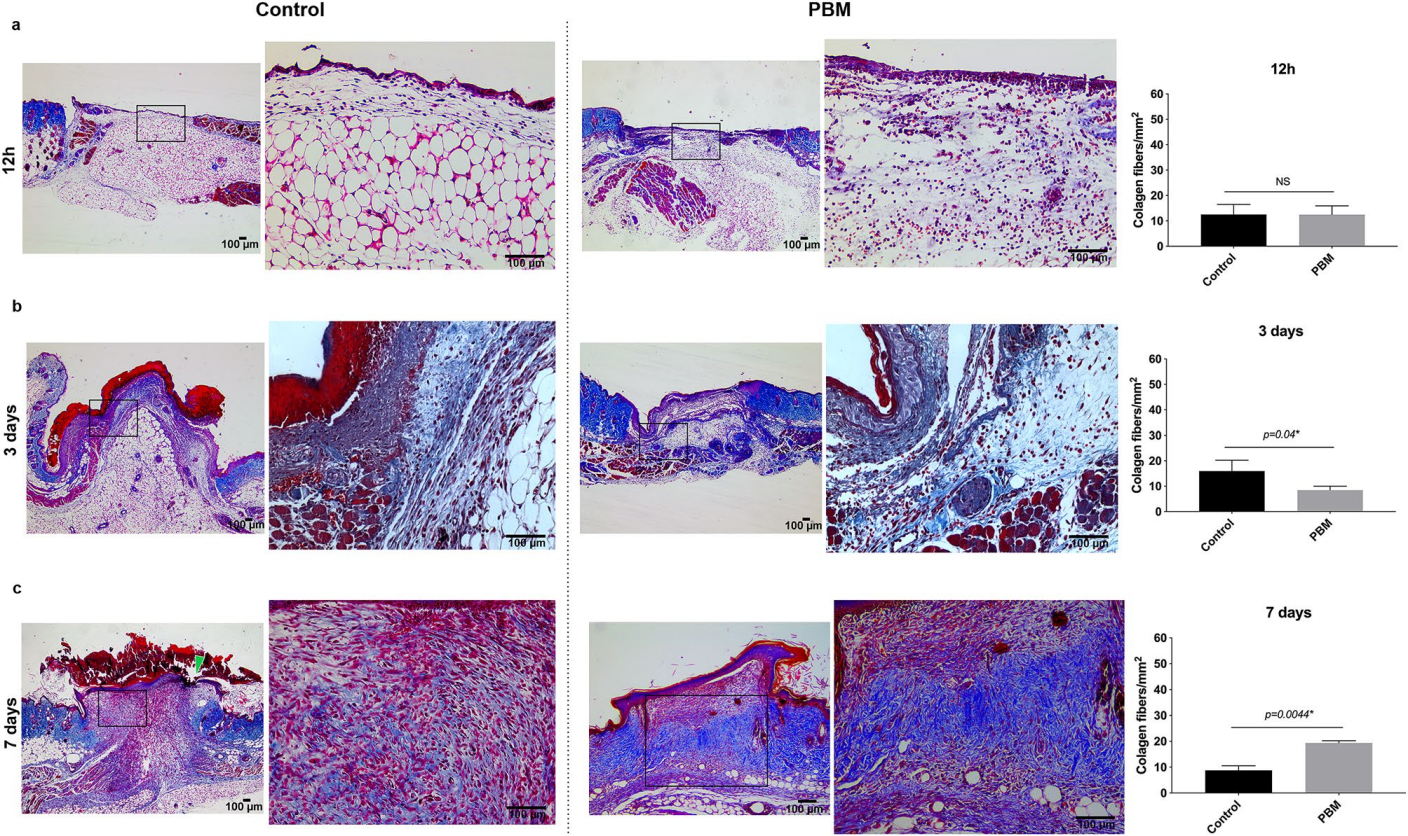
PBM effects on Type I collagen fibers deposition. Masson trichrome stain in the control and PBM-treated wounds at experimental times 12 h (**a**), 3 days (**b**), and 7-days interval (**c**). The squared region is depicted in higher magnification (× 200) in the following image. The green arrow ((**c**), first image) points to an ulcerative region in the control wound. Bar graphs demonstrate the comparison of collagen fibers quantification between the PBM-treated and control wounds. Data are shown as the mean ± SD, n = 5 for each group. *N.S.* non-significant. Scale bar = 100 µm.

At the 12 h time interval, the mean score of fibrin clot formation and angiogenesis degree were similar between both groups (*p* = 0.664) (Sup. Fig. S5). However, the inflammatory infiltrate was significantly increased in the photoactivated wounds than in the controls (*p* = 0.019) (Sup. Fig. S5). At 12 h, no difference in mast cell count was observed between PBM-treated and control wounds (*p* = 0.164) (Fig. 5a,b). Despite this, PBM led to a significant reduction of eosinophil counts in this early inflammatory phase of wound repair (*p* = 0.020) (Fig. 5c,d). No differences concerning collagen fibers deposition were observed (*p* = 0.986) (Fig. 6a).

**Figure 7:**
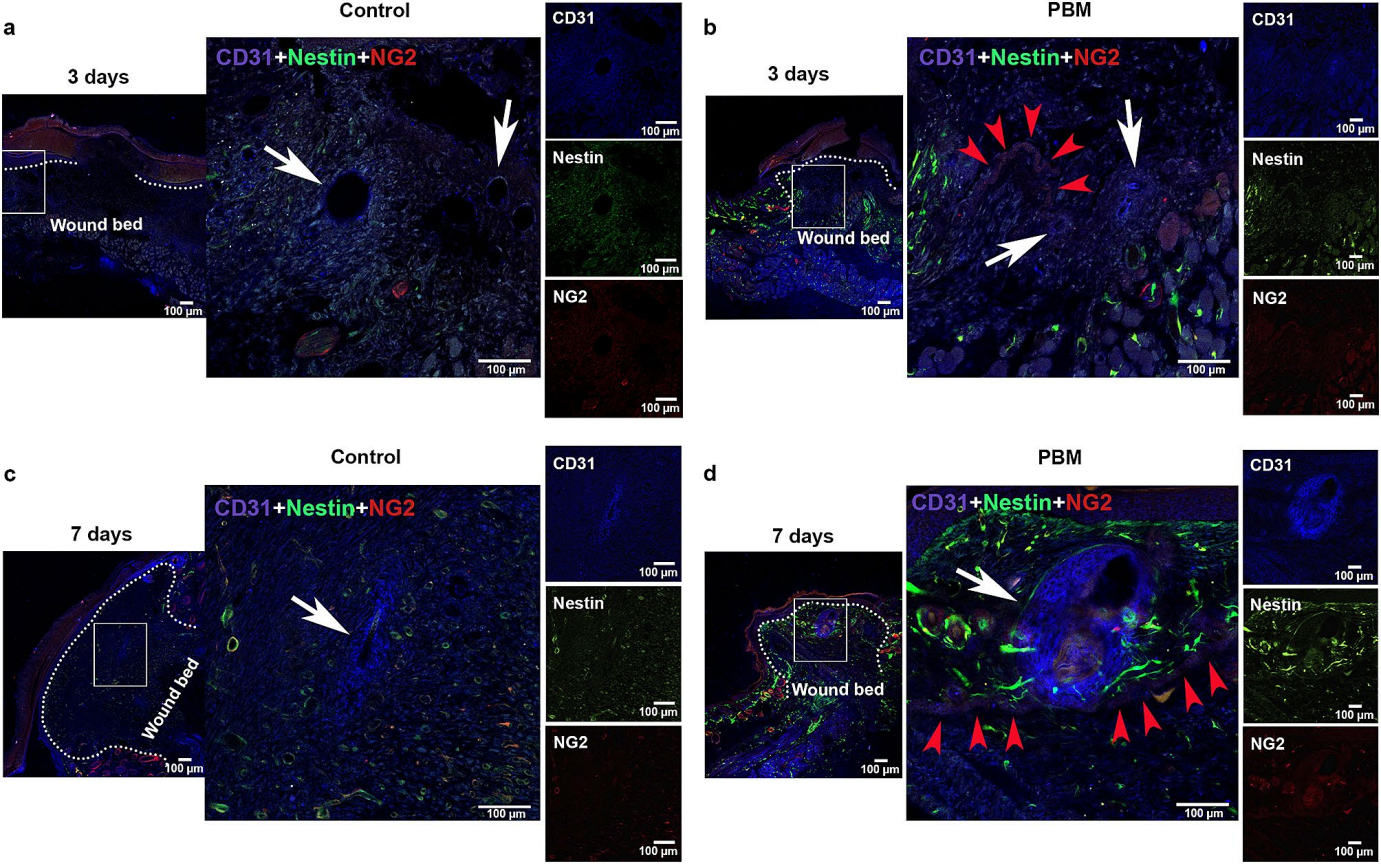
PBM effects on the skin microvasculature. CD31 marker epifluorescence images of the immunostaining for control and PBM-treated wounds at day 3 (**a**,**b**) and 7 post-surgery (**c**,**d**). In all groups and experimental times, the squared region is depicted in higher magnification (× 200) in the following image. PECAM-1 positive vessels (white arrows) are observed in the control (**a**,**c**) and in the PBM-treated samples (**b**,**d**) in both experimental times. Note the presence of an arteriole in a representative PBM sample (**d**). Also, note small capillaries in the photoactivated samples where the NG2 protein is observed colocalized with the CD31 marker (red arrowheads) (**b**,**d**). Scale bar = 100 µm.

At day 3 time interval, the epidermis area and thickness were significantly higher in the control group compared to the photoactivated wounds showing a premature but disorganized epithelialization process (*p* = 0.012 *and p* = 0.009*,* respectively) (Sup. Fig. S6). In the PBM-treated groups, the epithelium was thinner but continuous (Sup. Fig. S6a). The granulation tissue area was also higher in control concerning the PBM-treated wounds (*p* = 0.022) (Sup. Fig. S6b). Otherwise, the scores for the degree of angiogenesis (where blood vessels were defined as a tube-like structure with a lumen, whether or not it contained red blood cells) were significantly higher in the photoactivated wounds than in the controls (*p* = 0.033) (Sup. Fig. S6b). CD31 staining has shown vessels in the controls mostly located at the wound edge (Fig. 7a). Small caliber vessels showing the expression of the CD31 marker or a colocalization of the NG2 and CD31 were identified in the photoactivated samples’ wound bed after 3 days of treatment (Fig. 7a). No differences between groups were observed for the mean score of fibrin clot formation (*p* = 0.864), epidermal differentiation (*p* = 0.880), and in the amount of inflammatory infiltrate (*p* = 0.851) (Sup. Fig. S6b). Eosinophil counts were similar in both groups (*p* = 0.421) (Fig. 5d). However, a higher number of mast cells were found in the PBM-treated wounds after 3 days (*p* = 0.031) (Fig. 5b). Signs of dermis reconstruction characterized by the deposition and reorganization of collagen fibers were observed mostly in the control group than in the PBM-treated wounds (*p* = 0.040) (Fig. 6b).

**Figure 8:**
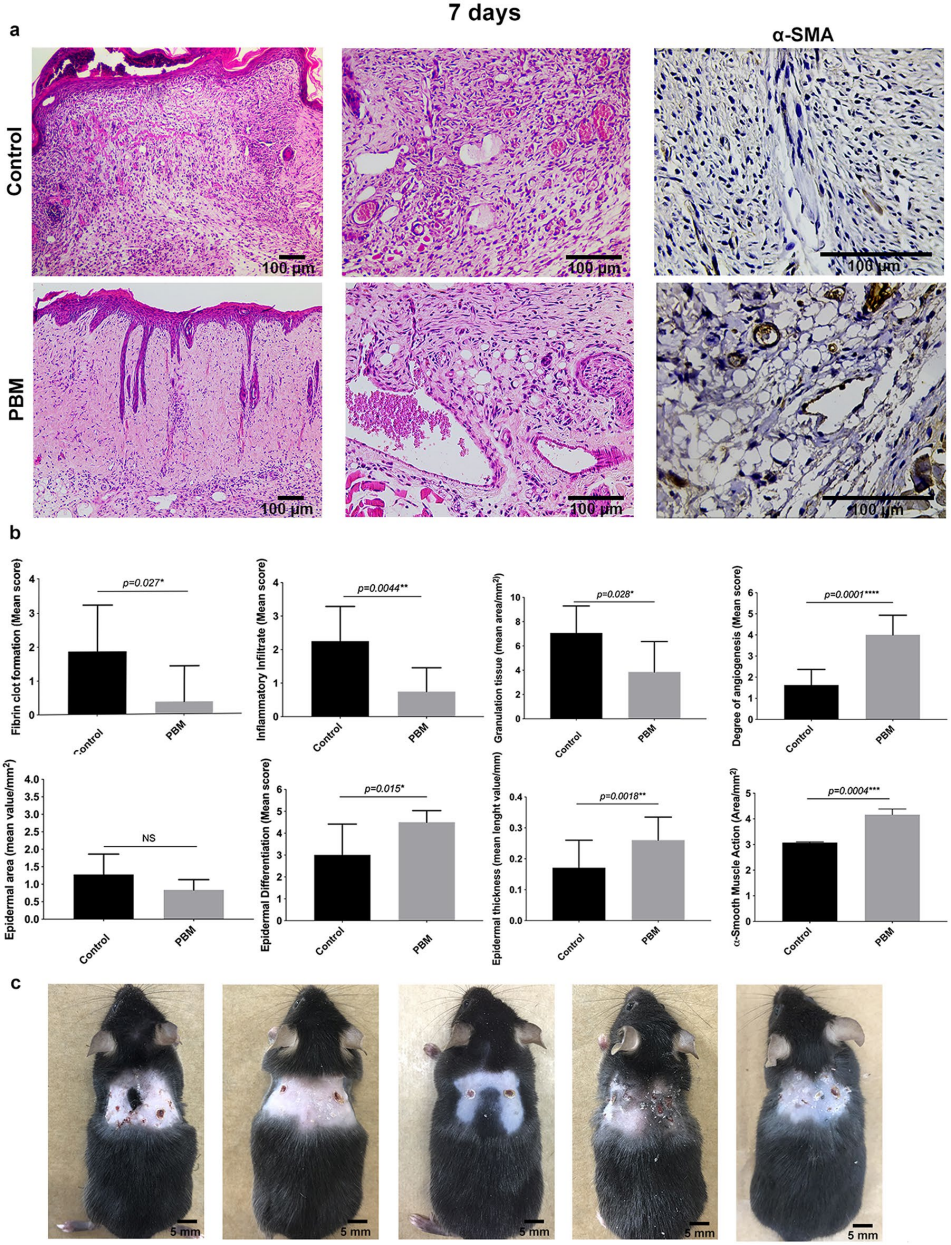
Histological evaluation of wound healing at day 7 post-surgery. Representative H&E-stained sections of control and PBM-treated wounds (**a**). Representative α-SMA immunostaining for control and PBM-treated wounds showing greater positive α-SMA cells around small blood vessels (**a**). Bar graphs demonstrate the comparison of the assessed histological parameters between control and PBM-treated wounds (**b**). Note that PBM-treated wounds present more advanced stages of epithelial maturation, increased vascularization and less inflammation and granulation tissue formation (**b**). Macroscopy of the wounds at day 7 post-surgery: control (right side) and PBM-treated (left side) wounds comparing both sides within the same animal (n = 5) (**c**). Data are shown as the mean ± SD, n = 5 for each group. *N.S.* non-significant. Scale bar = 100 µm (photomicrographs) and 5 mm (macroscopy).

After 7 days of treatment, histological analysis and macroscopy have indicated that PBM-treated wounds presented more advanced tissue repair stages than the control (Fig. 8). PBM-treated wounds have shown more blood vessels, including larger caliber vessels and vascular congestion, than those observed in the control group (*p* < 0.001) (Fig. 8b). CD31 staining has shown few vessels within the wound bed of the controls (Fig. 7c), while an exuberant arteriole and small caliber vessels could be identified in the photoactivated samples (Fig. 7d). Once again the colocalization between the NG2 and CD31 marker could be observed in vessel structures (Fig. 7d). The granulation tissue area was significantly smaller than in the control wounds (*p* = 0.028) (Fig. 8b). At this time point, fibrin clot formation and inflammatory infiltrate were reduced considerably in the PBM-treated wounds than in the controls (*p* = 0.027 and *p* = 0.004, respectively) (Fig. 8b). Eosinophils quantification was similar in both groups (*p* = 0.404) (Fig. 5d). However, the number of mast cells was still higher in the PBM-treated samples than in the controls (*p* = 0.004) (Fig. 5b). PBM-treated wounds presented more significant epidermal differentiation (*p* = 0.015) and epithelium thickness (*p* = 0.001), clearly showing granular and spinous layers of the regenerated epithelium (Fig. 8b). Epidermal indentation could also be detected in some of the PBM-treated samples but not in the controls (Fig. 8a). As such, the control wounds have shown more prevalence of the basal layer, which indicates a more proliferative status of the epithelium (Fig. 8a). Moreover, even on the 7th day, fragmented epithelium showing ulcerative regions could be noted only in the control groups (Fig. 6c).

The Masson trichrome mostly stains type I collagen fibers. Accordingly, increased collagen fibers distribution, packing, and orientation were observed in the PBM-treated groups on day 7th compared to the controls (*p* = 0.004) (Fig. 6c). A higher number of cells expressing α-smooth muscle actin (α-SMA)—which characterizes the myofibroblast phenotype—were seen around blood vessels but also as isolated cells dispersed within the interstitial tissue, particularly in the PBM-treated wounds (*p* < 0.001) (Fig. 8a,b).

Together, these data indicate that photobiomodulation significantly accelerates tissue repair through its vascular effects with direct recruitment of pericytes to the injury site (Sup. Fig. S7).

## Discussion

The absorption of visible light and infrared radiation may cause biological effects on the skin^25^. However, the beneficial mechanisms of light exposure have not been fully elucidated. In this study, we have investigated the ability of PBM at 660 nm in driving endogenous cells to collaborate in tissue repair. Using a reporter model for pericytes, our study has demonstrated that PBM promotes remarked vascular support and significantly accelerates the skin repair through an overall cellular input scenario with the direct involvement of Type-2 pericytes.

The association of pericytes and PBM in the skin was only barely explored. Using immunohistochemical techniques, Medrado et al. showed an increased number of NG2^+^ and α-SMA^+^ cells—both well-recognized markers for pericyte cell targeting—in the PBM-treated groups with dosimetry parameters similar to the ones of the current study^26^. These cells showed a parabasal position, and some of them seemed to be detaching from the periphery of capillaries and venules when stimulated by PBM^26,27^. There is also accumulated evidence showing that PBM at 660 nm can increase local blood circulation^6,28,29^. In the present study, we have observed that, under the PBM stimulus, not only Type-2 pericytes but also undifferentiated cells (Nestin^+^) identified by confocal microscopy were clearly attracted to the wound dermis upper region, close to the epidermis. Many of these cells seemed to be accumulating in or detaching from blood vessels dispersed into the dermis region, particularly at the wound healing process’s early stages. This is the first time that these cells’ colocalization is shown in the injury site of photoactivated wounds using state-of-art techniques. However, it remains unclear whether PBM contributes to the direct activation of pericytes and endothelial cells or is indirectly responsible by the recruitment of these and other cells due to the marked vascular changes promoted by the light.

A particular immune component of the wound healing process is the mast cell (MC). MCs represent up to 8% of the total number of cells within the dermis and are known to infiltrate at neovascularization sites at the beginning of the wound healing process^30,31^. In truth, the MCs topographic distribution within small vessels' vicinity guarantees provisions of the essential mediators needed for normal angiogenesis, such as histamine, serotonin, tumoral necrosis factor-α, kinins, and tryptases/proteases^32,33^. Herein, we have quantified Toluidine Blue O-stained MCs population in the PBM-treated groups, both intact or degranulated, and observed a progressive increase in MCs counts compared to the controls throughout the experimental times (12 h, 3 and 7 days). In this line, a previous study has also found a significant increase in MCs count after 3 days of injury in wounds treated with PBM and a concomitantly higher expression of vascular endothelial growth factor—a potent vasoactive growth factor^34^. Others have shown that red and infrared wavelengths promote immediate MCs’ degranulation in the human gingival tissue^35,36^. Recently, Kimizuka et al*.* has demonstrated that the increased microvascular permeability induced by PBM is mediated by MCs^6^. Our findings are in line with the current literature, which partially explains the acceleration of the tissue repair in the photoactivated tissues as a consequence of MCs degranulation leading to the early increase of vascular permeability.

PBM treatment is known to stimulate the transient receptor potential vanilloid 1, 2, and 4 present in the plasmatic membrane of MCs^9,37–39^. PBM-activation of transient receptor potential vanilloid-2, for example, allows Ca^2+^ ions to enter the cell, thus feedbacking MCs degranulation^37,38^. However, in 2005, Tóth et al*.* demonstrated by immunoelectron microscopy that brain pericytes also present transient receptor potential vanilloid-1^40^. This knowledge can contribute to the understanding regarding pericytes recruitment during PBM stimuli in the skin healing or repair. Accordingly, increased blood flow and increased angiogenesis observed in the PBM-treated wounds on the 3rd and 7th day may correlate to the sustained higher number of MCs during almost all experimental times, and with the consequent cellular input to the injury site. Nevertheless, the hypothesis of direct activation of pericytes by PBM is also reasonable once, in addition to physically stabilize blood vessels and contribute to the vascular development, maturation and remodeling, pericytes may regulate the permeability and the blood flow^41^. Further work is needed to explore the crosstalk between pericytes and mast cells under the PBM scenario considering their shared vascular role.

Considering that MCs and eosinophil cells are developmentally and functionally similar, we also have looked for eosinophil cells participation in the wound healing process. We have observed a significant suppression in the eosinophils count in the first 12 h after PBM. After 3 and 7 days, these cells’ counts were restored but slightly greater in the PBM-treated than in the control groups. Eosinophils are, in fact, important to degrade collagen, but they also stimulate dermal fibroblast DNA synthesis and matrix production^42^. However, MCs and eosinophil cells can be influenced by paracrine mechanisms. For instance, for eosinophils, histamine may act as a chemoattractant through histamine receptor (H1R)^43^ and inhibit chemotaxis through histamine receptor (H2R), i.e., when histamine concentration is high^44^. Although our results do not fully support the true interaction between MCs and eosinophil cells under the PBM influence, the inverse ratio of these cells may be contributing to the premature collagen deposition in the controls (on the 3rd-day time interval) and to the belated collagen deposition in the PBM-treated wounds (on the 7th-day time interval).

A light on the PBM's influence in the process of epithelialization of the wounds is worth noting. We have observed that PBM-treated groups presented more continuous and differentiated epithelialization, but only at the 7th day-time interval. Earlier, at the 12 h and 3rd-day intervals, the epithelium was thinner, and the wounds presented macroscopically humid. Despite it, secondary infection was found in none of the groups. More recently, it has been demonstrated that infrared laser induces unique immunologic cascade in the MCs via reactive oxygen species generation and creates an immunostimulatory milieu for dendritic cells, augmenting the adaptive immune response and playing a critical role in the host defense^6^. We hypothesize that PBM-treated groups presented a gradual keratinocyte epithelialization process, which may have allowed enough time to connective tissue reconstruction and remodeling.

In the current study, the PBM-treated groups kept expressively vascularized after 7 days, and less granulation tissue was formed. The myofibroblast population in the PBM-treated groups was increased compared to the controls, as represented by the marked expression of α-SMA. Macroscopically, these groups presented thinner eschars and were more uniformly healed in comparison to the controls. Indeed, tissue regeneration harmoniously progressed toward the center of the wound in most of the PBM-treated samples. Notably, in the PBM-treated groups at the 7th day time interval, samples were repaired almost entirely, and some were presenting restored skin attachments, such as the hair follicles. As aforementioned, together with undifferentiated cells, Type-2 pericytes have accumulated in the upper region of the dermis of PBM-treated wounds. Curiously, despite numerous of these cells seemed to be detaching from blood vessels, we have also noticed some yellowish and pinkish cells around the hair follicles, particularly in the wound edges from both groups. The skin and its appendages undergo continuous renewal and maintain reservoirs of multipotent stem cells that are promptly activated upon skin injury^45^. A classic stem cell niche is localized in the bulge region of the hair bulb, and after wounding, these cells give rise to the epidermis, follicles, and sebaceous glands^46^. Even though the NG2 protein can be expressed by hair follicles cells^47,48^, the association of these cells' pericytic origin needs further attention.

One of the mainstream regulators of PBM-induced cascades is the Transforming Growth Factor-β1, which is directly activated from its latent state by light^2,3^. Besides many other functions, Transforming Growth Factor-β induces chemotaxis of inflammatory and immune cells to the wound site, drives stem cell differentiation, and mediates collagen type III replacement with collagen type I^49^. Recently, the study of Parfejevs et al. showed that injury-activated glia cells promote myofibroblast differentiation by paracrine modulation of Transforming Growth Factor-β signalling in wound healing^50^. Pericytes development and survival are also regulated by Transforming Growth Factor-β1, although many other signals can participate^51^. The association of the increased number of undifferentiated cells and Type-2 pericytes with the marked vascular changes can be critical elements involved in the PBM therapy process, thus contributing to the structural replenishment of the wounds very close to the original skin.

Finally, the present study model has limitations concerning the Nestin^+^ cells' identity once green fluorescent protein levels may not accurately mirror the *Nestin* gene activity^52^. Otherwise, our transgenic mice lineage may be considered one of the most appropriate animal models to understand the molecular and cellular components involved in the PBM-triggered cascades. Noteworthy, the study was performed with test and control groups in the same animal due the phenotype's rarity. Although there is an appeal that PBM may have a systemic effect, we have observed that the local stimulus is critical. Future studies should investigate the mechanisms underlying pericytes angiogenic potential under photoactivation and whether their ablation affects angiogenesis and PBM outcomes.

## Materials and methods

### Ethical issues and animal procedures

The Animal Care and Use Committee at Universidade Federal de Minas Gerais approved animal handling and procedures in this work (#19/2018), and all experiments were performed according to the ARRIVE guidelines. Our Nestin-GFP^+^ transgenic mice colony was maintained homozygous for the transgene on the C57BL/6 genetic background^23^. Nestin-GFP^+^ mice were crossbred with NG2-DsRed^+^ mice to generate Nestin-GFP^+^/NG2-DsRed^+^ double-transgenic mice. Accordingly, this model presents endogen fluorescence for two proteins: Nestin-GFP^+^/undifferentiated cells (marked in green) and NG2-DsRed^+^/pericytes (marked in red). All animals were housed in controlled conditions for temperature (24 °C) and under a 12:12-h light–dark cycle and fed ad libitum.

Eight to ten-week-old male or female Nestin-GFP^+^/NG2-DsRed^+^ mice (n = 15; 5 per group) were used in this study. Animals were anesthetized [100 mg/kg (Ketamine) + 10 mg/kg (Xylazine) via intraperitoneal—IP] and two full-thickness excisions (including the *panniculus carnosus*) were performed on the back of the mouse, one on each side of the animal midline, by using a 4.0 mm biopsy punch. A 0.5 mm thick silicone splint was then placed around the wounds and sutured with six 5.0 nylon stitches to prevent premature wound closure^53^ (Sup. Fig. S1). Next, the left wound received the PBM treatment, while the other was kept as the control.

### Photobiomodulation setting parameters

PBM was performed by using an indium–gallium–aluminum–phosphide (InGaAlP) customized diode laser, in continuous operation and punctual and contact modes. The dosimetry parameters were as follows: 660 nm, 20 mW, 0.028 cm^2^ spot area, 0.71 W/cm^2^, 7 s, 5 J/cm^2^, 0.14 J total energy per point^5^. The photoactivation was performed transoperatively and every day up to 7 days (Sup. Fig. S1). Noteworthy, unless wound measurements needed to be done, animals were only manually restrained for the photoactivations. PBM exposure was avoided in the control wound by using a black cardboard paper over it. The output power was checked with a power meter (Lasercheck, Coherent Inc., Santa Clara, California, USA) before all photoactivations. A double microporous tape (Nexcare, 3 M, Saint Paul, Minnesota, USA) was then applied on the silicon splint as an occlusive patch and replaced every day to minimize wound dehydration in both groups. Each mouse was individually caged to prevent stitches biting.

### Confocal intravital microscopy

In vivo imaging of Nestin GFP/NG2 DsRed double-transgenic mice under anesthesia (100 mg/kg ketamine and 10 mg/kg xylazine, IP) at time intervals of 12 h, 36 h and 72 h were performed by using an Eclipse Ti with an A1R confocal head (Nikon, Tokyo, Japan) equipped with four different lasers (excitation at four wavelengths: 405, 488, 546 and 647 nm) and emission bandpass filters at 450/50, 515/30, 584/50 and 663/738 nm^54,55^. Objective Plan Apo 20 × was used. Undifferentiated cells (Nestin^+^), total pericytes population (NG2^+^) and Type-2 pericytes subset (Nestin^+^, NG2^+^ and Dapi colocalization shown in yellow) were counted by the fluorescence intensity (mean gray values) using the Fiji software (NIH, Bethesda, Maryland, USA).

### Blood flow analysis

Blood flow at the wound sites was assessed by Laser Doppler Perfusion Imaging (MoorLID2-IR, Moor Instruments, Devon, UK) under anesthesia (100 mg/kg ketamine and 10 mg/kg xylazine, IP) at 3 and 6 days after the first photoactivation (Sup. Fig. S1f). The mean pixel value of each scanned image was calculated using the Moor LDI V5.3 software, and expressed as perfusion units, representing the average blood flow at the wound sites.

### Macroscopy

Under anesthesia following previously cited conditions, wounds were measured according to Moreira et al*.* on days 2, 3, 4, 6 and 7 by using a digital pachymeter (Mitutoyo, Kawasaki, Japan) and digital images were acquired from each animal up to the 7th day^56^.

### Histological assessment

The mice were euthanized after 12 h, 3, and 7 days (Sup. Fig. S1f). Samples were collected 2.0 mm beyond the wounds and fixed in paraformaldehyde 4% for 48 h and processed for both optimal cutting temperature compound or paraffin embedding. Optimal cutting temperature compound was done in 20 µm cryosections (CM3050 S, Leica Microsystems, Morrisville, North Carolina, USA), and the slides were observed by confocal microscopy. Paraffin blocks were serially sectioned using a microtome at 6 μm sections (Leica). Five consecutive histological fields in five different sections were selected to analyze re-epithelialization and inflammatory process grading (40 × or 100 ×). According to a previous study^57^, the inflammatory process intensity, fibrin clot formation, degree of angiogenesis, epithelium differentiation, and indentation were given a score of 0 to 5 based on its level of abundance in the wound beds according to a previous study. The granulation tissue area, epithelium area and thickness were quantified by the Fiji software (NIH). All samples were analyzed with an optical microscope (Axioskop 40 Zeiss, Göttingen, Lower Saxony, Germany), and one blinded examiner undertook all histological analysis. Detailed staining protocols are described below.

### Masson's trichrome stain for collagen fibers and quantification

Sections were stained with Harris hematoxylin (Newprov, Pinhais, Paraná, Brazil) for 40 s, washed under running tap water for 2 min and stained with Masson's trichome solution for 17 min. Images of consecutive regions of the wound area were taken in × 200 magnification using an optic microscope (Axioskop 40 Zeiss; Carl Zeiss). The identification of collagen fibers was made by deconvolution of color images on ImageJ software Version 1.52 (NIH, Bethesda, Maryland, USA). During color image deconvolution, images with Masson’s trichrome stain were deconvolved, and the green component was identified as the collagen fibers. The area of the green collagen fibers was measured using the “threshold” tool. The threshold was manually adjusted until the entire green area was highlighted in red. Then, the threshold was measured as the percentage of the stained area^58^.

### Toluidine blue staining for mast cell identification

Sections were stained with 1% Toluidine Blue O (89640, Sigma Aldrich, Saint Louis, Missouri, USA) in distilled water for 2 min, to the identification of mast cells^59^. For each histological section, five consecutive histological fields at × 400 magnification were selected for the analysis. These fields were located in the dermis and submucosa regions of the wound beds. Mast cell counting was performed according to Sawasaki et al. and was blindly performed by the same examiner^36^.

### Sirius red staining for eosinophil identification and quantification

Sirius red staining is a method used to access the presence of eosinophils^60^. Briefly, slides were incubated in Harris hematoxylin for 2 min, rinsed in tap water, and then 100% ethanol. Subsequently, slides were immersed in an alkaline (pH 8–9) Sirius Red solution (CI 35780, Sigma Aldrich) for 2 h. Ten random foci were selected from low power in an optic microscope (Axioskop 40 Zeiss; Carl Zeiss) and were assessed by one blinded examiner. Eosinophils were counted per viewing field (× 400 magnification) and averaged for each wound bed.

### α-Smooth muscle actin (α-SMA) immunohistochemical analysis

Immunohistochemistry was performed for α-SMA detection at day 7 post-surgery in the wound sections. Briefly, the slides were deparaffinized and dehydrated, followed by incubation with 3% hydrogen peroxide and 1% bovine serum albumin (BSA). The sections were then incubated with a polyclonal rabbit anti-mouse α-SMA primary antibody (MM1, Novocastra, Newcastle, UK, 1:200) at room temperature for two hours. The immunolabeling was visualized through incubation in 3,3-diaminobenzidine (DAB) solution (Dako, Carpinteria, California, USA). Then, the sections were stained with Mayer’s hematoxylin and covered. Negative controls were obtained by omission of the primary antibody, which was substituted for 1% PBS-BSA. Images of 10 consecutive fields were taken in × 200 magnification using an optic microscope (Axioskop 40 ZEISS; Carl Zeiss, Gottingen, Germany). Immunostaining for α-SMA was quantified by color deconvolution plugin on ImageJ Version 1.52 (NIH). The red component was identified as α-SMA staining, and a threshold was manually adjusted and finally measured as the percentage of the stained area in the wound beds.

### CD31 immunofluorescence analysis

Immunofluorescence was performed for CD31 detection at day 3 and 7 post-surgery in the wound sections. Briefly, cryosectioned slides were rehydrated twice in PBS, followed by incubation with Triton X-100 0.5% bovine serum albumine 2% in PBS for 1 h and a half for permeabilization and blocking. The sections were then incubated with a monoclonal rat anti-mouse conjugated CD31 primary antibody (Brilliant Violet 421 nm, Abcam, Cambridge, UK, 1:100) overnight at 4 °C.

### Confocal analysis

Skin sections were counterstained with Dapi and mounted on slides using Fluoroshield Mounting Medium (Abcam) and examined under confocal microscopy (Nikon Eclipse Ti with an A1R confocal head). Slides were examined under objectives Plan Apo 10 × and 20 × . A large scan image was performed for each sample under the 10 × objective. Each channel was counted separately, except for Type-2 pericytes that were counted in the merged images. A threshold was manually adjusted, and the fluorescence intensity (mean gray values) was calculated using the Fiji software (NIH).

### Statistical analysis

Statistical significance between groups was calculated using paired (for the remaining wound area and Doppler analysis comparisons) or unpaired Student’s *t-*test or Mann–Whitney test (for image analysis) using the GraphPad Prism 8.0 Software (GraphPad Prism Version 8.0c for Mac, GraphPad Software, La Jolla, California, USA). Data were expressed as means ± standard deviation. The differences among the groups were considered significant when *p* < 0.05 in all experiments.

## Supplementary information

Supplementary Legends.

Supplementary Figure S1.

Supplementary Figure S2.

Supplementary Figure S3.

Supplementary Figure S4.

Supplementary Figure S5

Supplementary Figure S6.

Supplementary Figure S7.

## Data Availability

All data generated or analyzed during this study are included in this published article (and its Supplementary Information files).
