# Supplementary material for: Photobiomodulation drives pericyte mobilization towards skin regeneration
Source: Sci Rep. 2020 Nov 6;10:19257. doi: 10.1038/s41598-020-76243-7 (PMC7648092; doi:10.1038/s41598-020-76243-7)
Supplement: Supplementary file 1 — Supplementary Legends. [file 41598_2020_76243_MOESM1_ESM.docx]

**Photobiomodulation drives pericyte mobilization towards skin regeneration**

Isabella Bittencourt do Valle^1,2^; Pedro Henrique Dias Moura Prazeres^3^; Ricardo Alves Mesquita^2^; Tarcília Aparecida Silva^2^; Hortência Maciel de Castro Oliveira^4^; Pollyana Ribeiro Castro^5^; Iuri Dornelas Prates Freitas^1,6^; Sicília Rezende Oliveira^2^; Natália Aparecida Gomes^1^; Rafaela Férrer de Oliveira^1^; Larissa Fassarela Marquiore^1^; Soraia Macari^1^; Flávio Almeida do Amaral^7^; Humberto Jácome-Santos^1,2^; Lucíola Silva Barcelos^5^; Gustavo Batista Menezes^4^; Márcia Martins Marques^8^; Alexander Birbrair^3^; Ivana Márcia Alves Diniz^1^*

**SUPPLEMENTARY MATERIAL**

**Supplementary figures**

**Figure S1.** Experimental procedures and timeline. The skin was stretched upwards to allow the simultaneous excision of both sides **(a)**. Two full-thickness excisions (including the *panniculus carnosus*) were performed using a 4 mm biopsy punch **(b)**. A 0.5 mm thick silicone splint was placed around the wounds and sutured to prevent premature wound closure **(c)**. The photoactivation was performed transoperativelly and every day up to the 7^th^ postoperative day, always on the mice left side **(d)**. The mean percentage of the wound remaining area in each side was calculated considering the vertical and horizontal measurements **(e)**. Scale bar = 5 mm.

**Figure S2.** Intravital analysis at 1h and day 3 post-surgery. Intravital representative epifluorescence images of split and merged channels on the central area (wound bed) and edge (wound edge) of the wound at 1h post-surgery **(a)**. Representative epifluorescence images of split and merged channels on the edge of the wound at day 3 post-surgery **(b)**. Note that cells were no longer observed at the central superficial area of the wounds at day 3 post-surgery. Squared regions are depicted in higher magnification (200×) in the images immediately below. Scale bar = 100 µm.

**Figure S3.** Labeled cell localization and distribution in transversal sections at 12h post-surgery. Epifluorescence images of split and merged channels of PBM-treated and control wounds showing tissue morphology and labeled-cells identification in both groups. Scale bar = 100 µm.

**Figure S4.** Labeled cells in the hair follicles. Representative epifluorescence split and merged images of PBM-treated and control groups at days 3 **(a)** and 7 **(b)** post-surgery. Hair follicles show pinkish (NG2^+^ and DAPI colocalization) and yellowish (Nestin+, NG2^+^, and DAPI colocalization) fluorescence. Undifferentiated and NG2^+^ cells were observed around and, sometimes, seemed to be detaching (arrow) from the hair follicles (arrowheads). Squared regions are depicted in higher magnification (200×). Scale bar = 100 µm.

**Figure S5.** Histological evaluation of wound healing at 12h post-surgery. Representative H&E-stained sections of the full-thickness excisional wound of the PBM-treated and control wounds. The squared region is depicted in higher magnification (100×) in the following image **(a)**. Bar graphs demonstrate the comparison of the assessed histological parameters between PBM-treated and control wounds **(b)**. Data are shown as the mean ± SD, n= 5 for each group. N.S. = non-significant. Scale bar = 100 µm.

**Figure S6.** Histological evaluation of wound healing at day 3 post-surgery. Representative H&E-stained sections of the full-thickness excisional wound of the PBM-treated and control wounds **(a)**. Note that photoactivated wounds present thinner epidermal layer and smaller epidermal area, increased vascularization (*), and less granulation tissue than the control. Arrows point to ulcerative areas in the control wound. Bar graphs demonstrate the comparison of the assessed histological parameters between PBM-treated and control wounds **(b)**. Data are shown as the mean ± SD, n= 5 for each group. N.S. = non-significant. Scale bar = 100 µm.

**Figure S7.** Summary of the main results. PBM-treated and control wounds are graphically represented comparing differences in the epithelialization process, wound size, and cellular and vascular components between groups.
