# Supplementary figures and images for: Photobiomodulation drives pericyte mobilization towards skin regeneration
Source: Sci Rep. 2020 Nov 6;10:19257. doi: 10.1038/s41598-020-76243-7 (PMC7648092; doi:10.1038/s41598-020-76243-7)

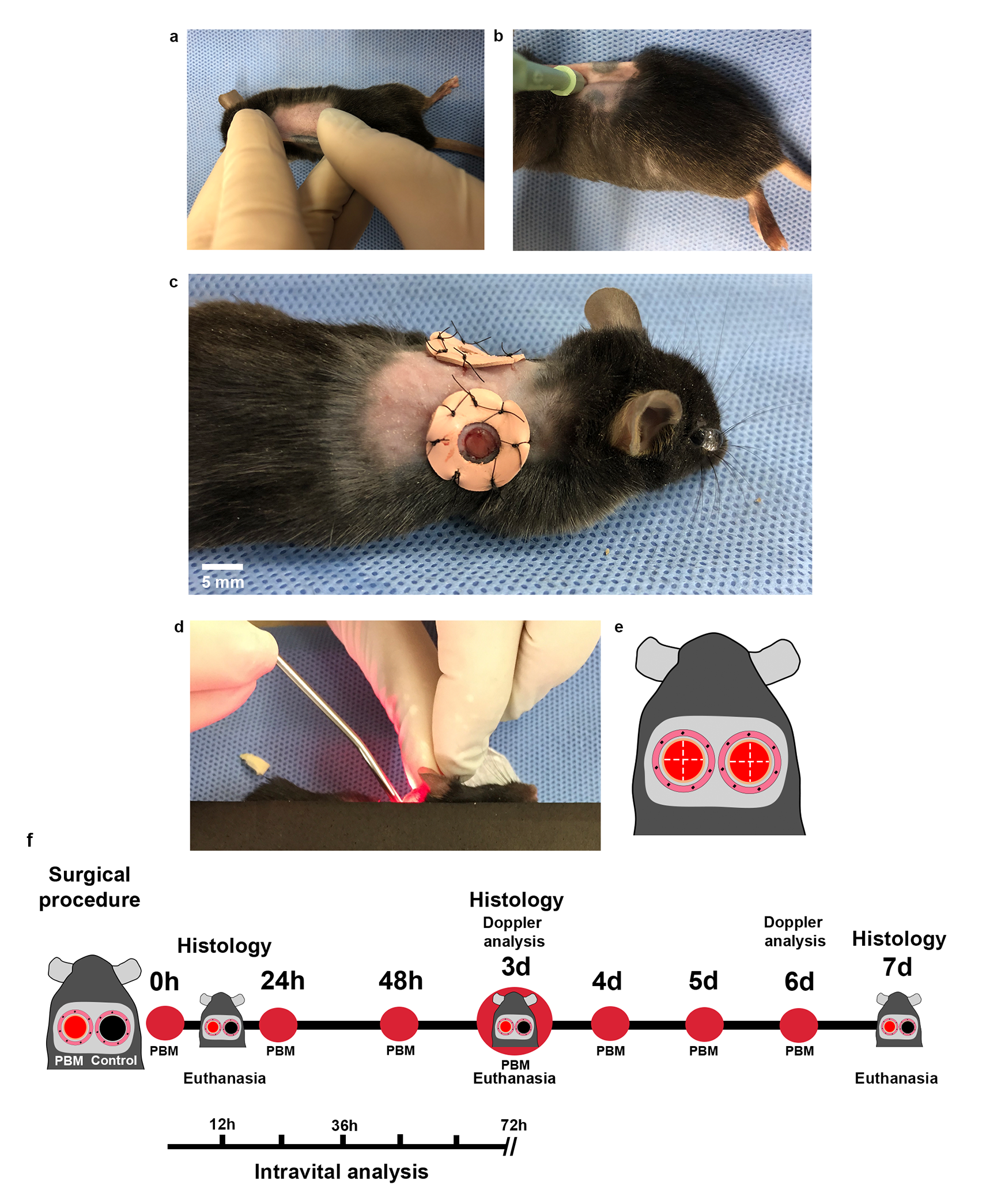

Supplement: Supplementary file 2 — Supplementary Figure S1. [file 41598_2020_76243_MOESM2_ESM.tif]

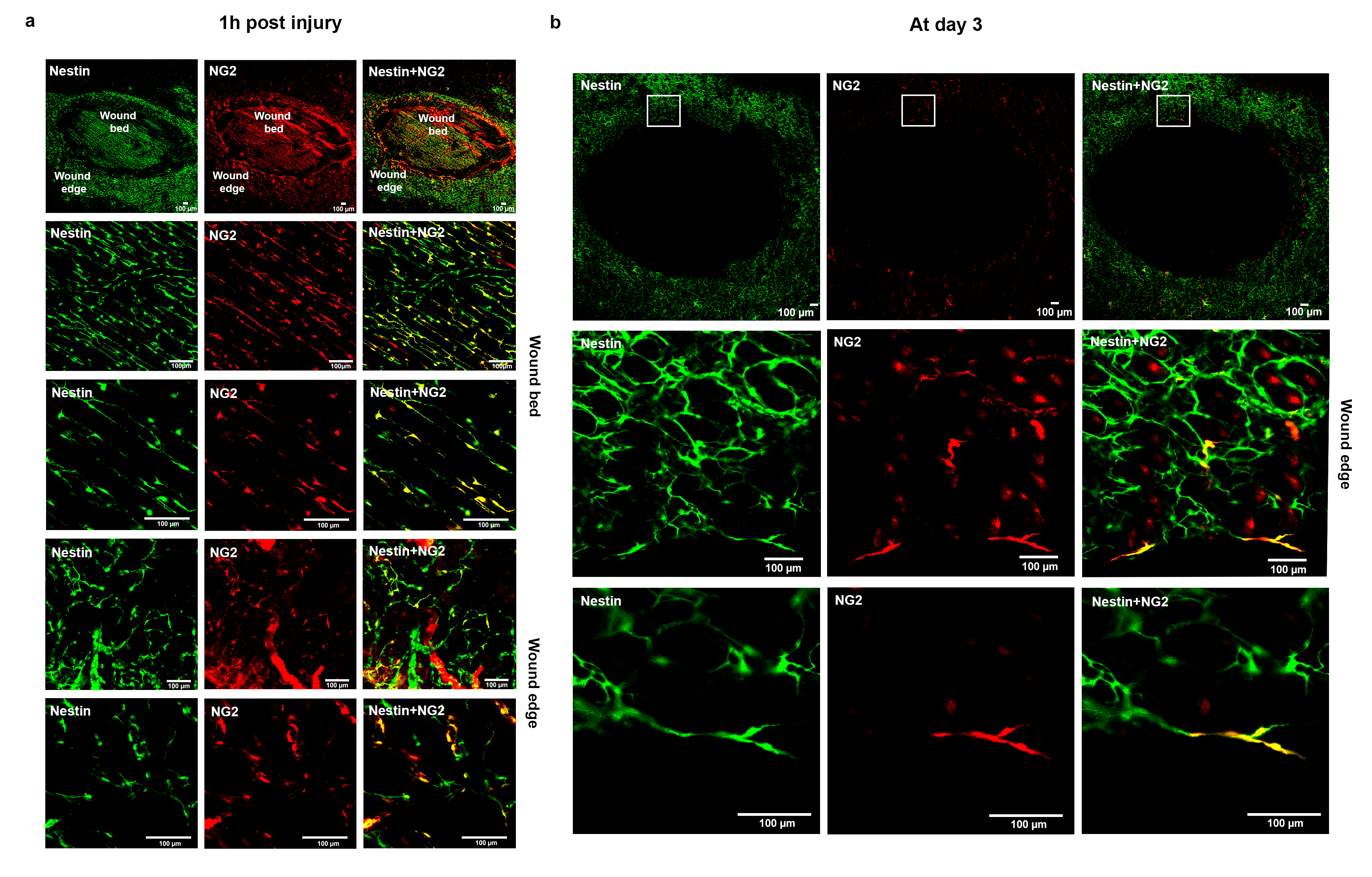

Supplement: Supplementary file 3 — Supplementary Figure S2. [file 41598_2020_76243_MOESM3_ESM.tif]

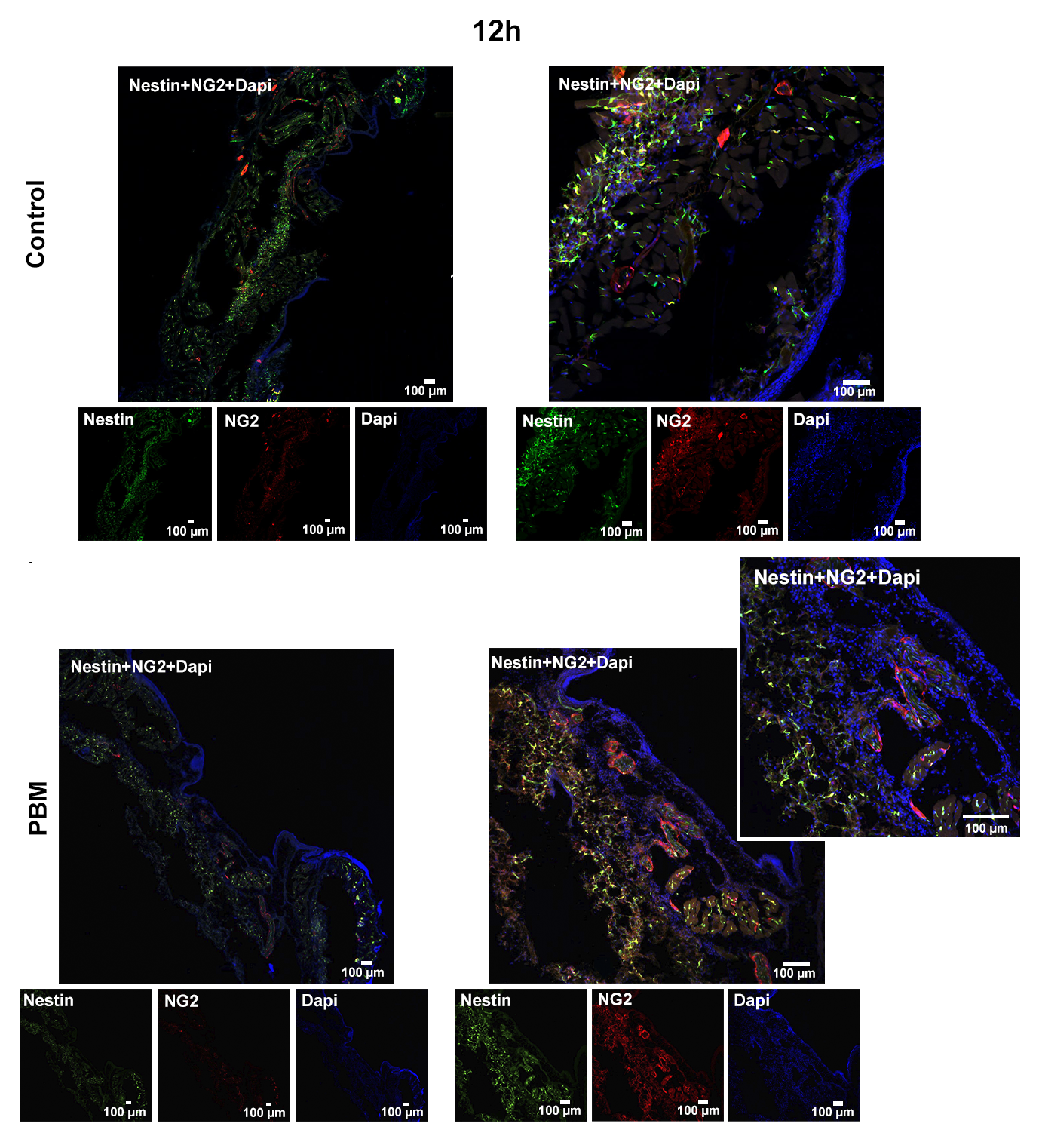

Supplement: Supplementary file 4 — Supplementary Figure S3. [file 41598_2020_76243_MOESM4_ESM.tif]

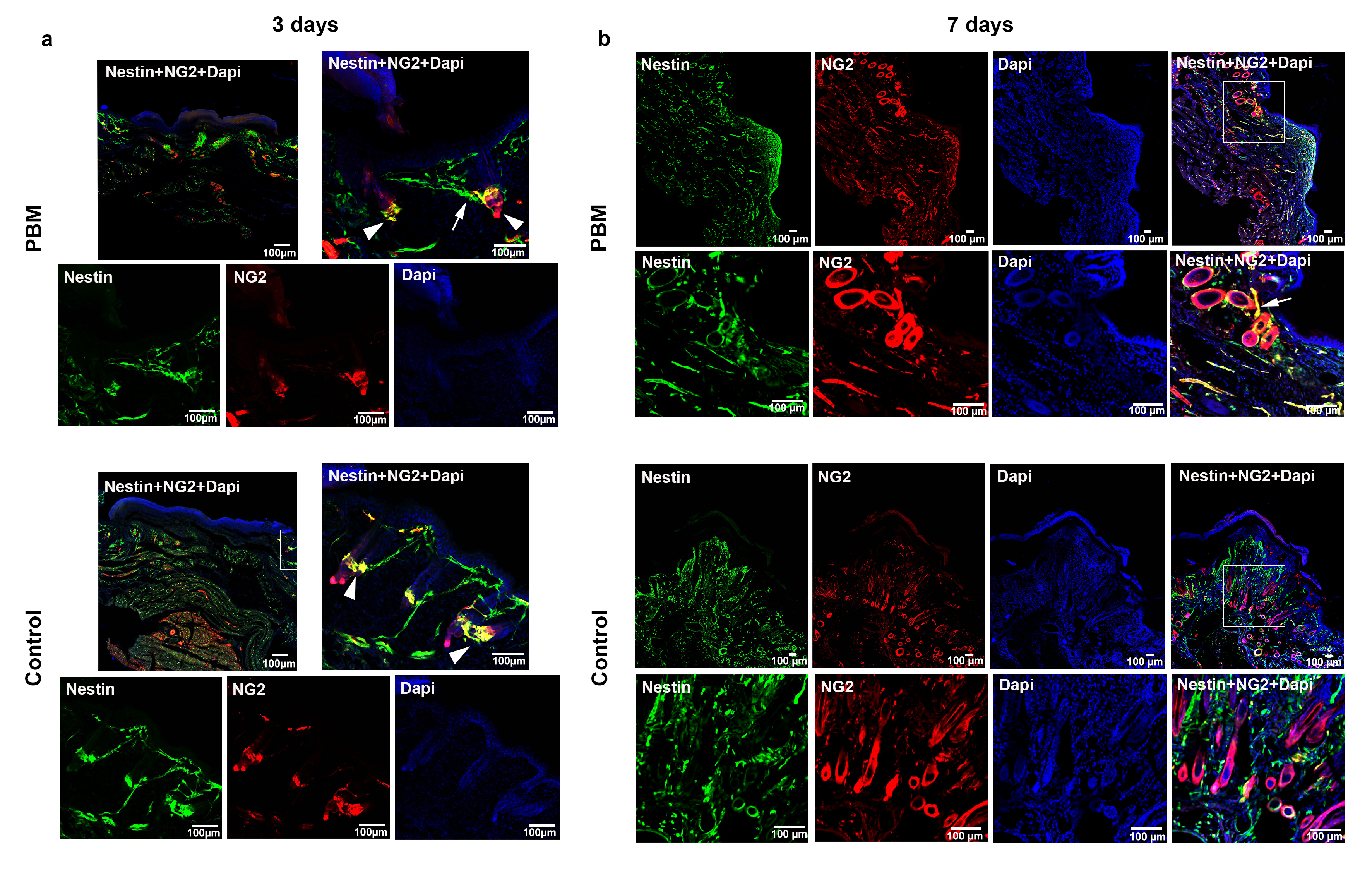

Supplement: Supplementary file 5 — Supplementary Figure S4. [file 41598_2020_76243_MOESM5_ESM.tif]

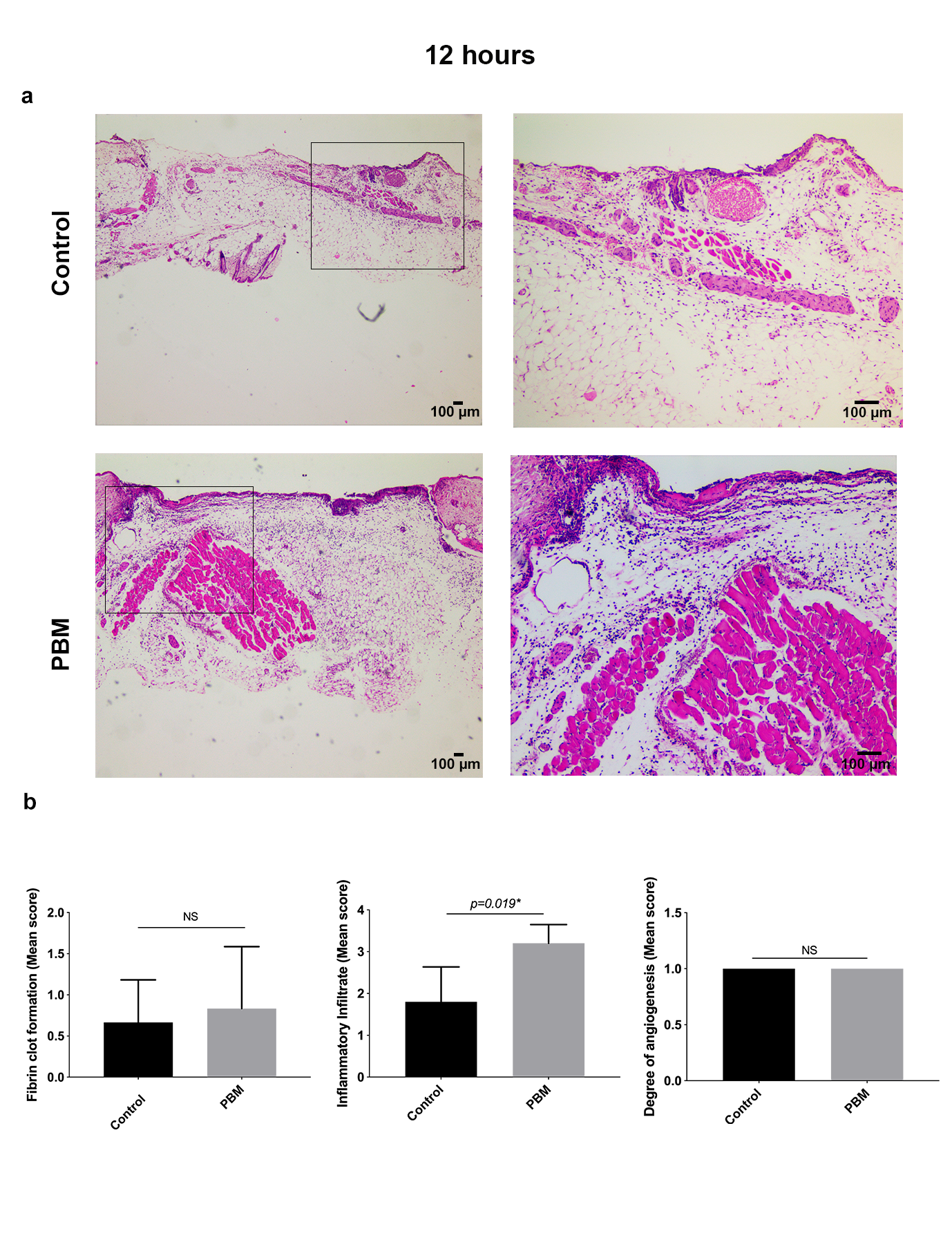

Supplement: Supplementary file 6 — Supplementary Figure S5 [file 41598_2020_76243_MOESM6_ESM.tif]

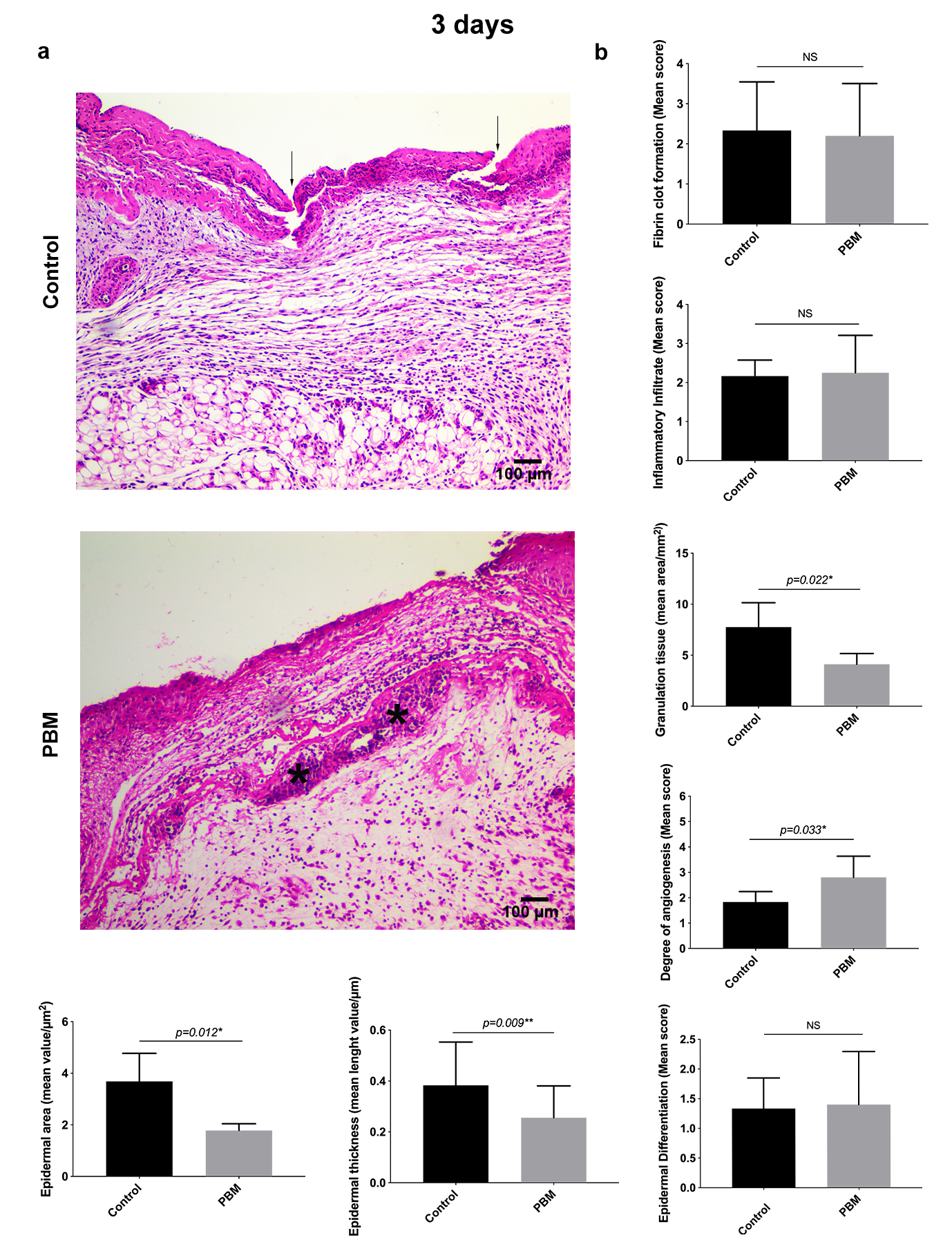

Supplement: Supplementary file 7 — Supplementary Figure S6. [file 41598_2020_76243_MOESM7_ESM.tif]

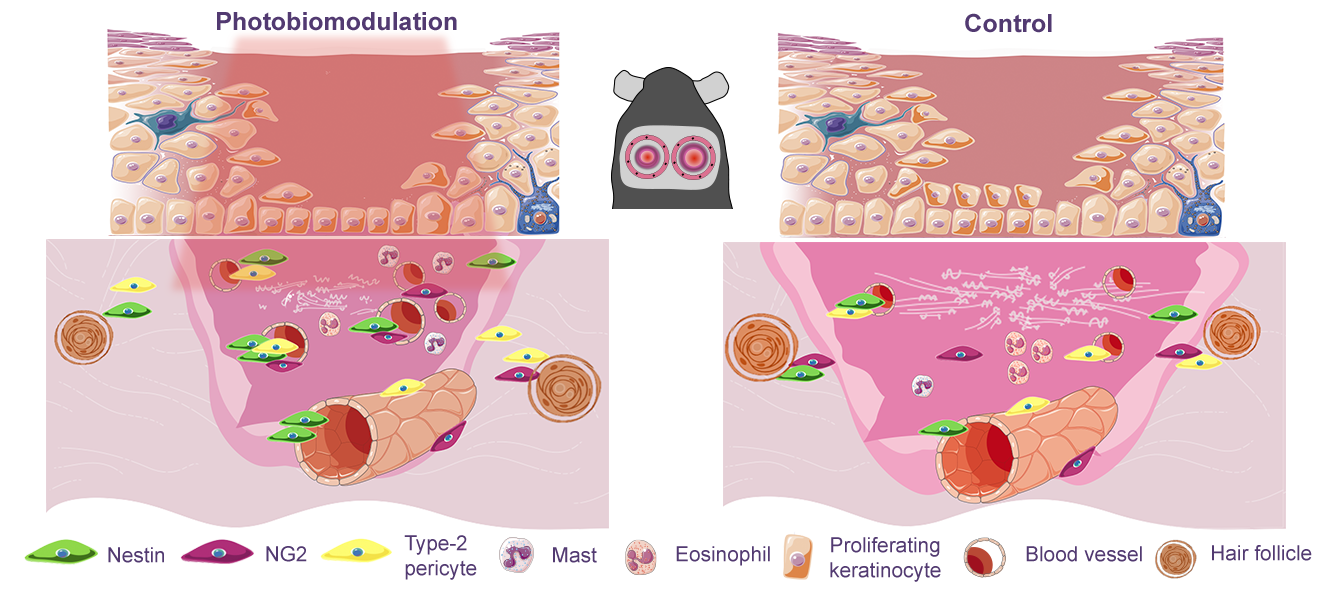

Supplement: Supplementary file 8 — Supplementary Figure S7. [file 41598_2020_76243_MOESM8_ESM.tif]
